# Evaluation of High Sensitive Troponin in Erectile Dysfunction

**DOI:** 10.1155/2015/548951

**Published:** 2015-04-16

**Authors:** Alessandra Barassi, Raffaele Pezzilli, Antonio Maria Morselli-Labate, Elena Dozio, Luca Massaccesi, Francesca Ghilardi, Clara Anna Linda Damele, Giovanni Maria Colpi, Gian Vico Melzi d'Eril, Massimiliano Marco Corsi Romanelli

**Affiliations:** ^1^Laboratorio di Analisi, Ospedale San Paolo, Dipartimento di Scienze della Salute, Università degli Studi di Milano, 20142 Milano, Italy; ^2^Dipartimento di Malattie dell'Apparato Digerente e Medicina Interna, Ospedale Sant'Orsola-Malpighi, Alma Mater Studiorum, Università degli Studi di Bologna, 40138 Bologna, Italy; ^3^Dipartimento di Scienze Biomediche per la Salute, Università degli Studi di Milano, 20133 Milano, Italy; ^4^Dipartimento di Scienze Biomediche, Chirurgiche e Odontoiatriche, Università di Milano, 20133 Milano, Italy; ^5^Istituto per la Sterilità e la Sessualità (ISES), 20123 Milano, Italy; ^6^Unità Operativa Medicina di Laboratorio-1 Patologia Clinica, IRCCS Policlinico San Donato, San Donato Milanese, 20097 Milano, Italy

## Abstract

*Background.* Evidence is accumulating in favour of a link between erectile dysfunction (ED) and coronary artery diseases. We investigated the presence of cardiac injury in patients who have had arteriogenic and nonarteriogenic ED using the hs-Tn levels. *Methods.* The diagnosis of ED was based on the International Index of Erectile Function 5-questionnaire (IIF-5) and patients were classified as arteriogenic (A-ED, *n* = 40), nonarteriogenic (NA-ED, *n* = 48), and borderline (BL-ED, *n* = 32) patients in relation to the results of echo-color-Doppler examination of cavernous arteries. The level of hs-TnT and hs-TnI was measured in 120 men with a history of ED of less than one year with no clinical evidence of cardiac ischemic disease. *Results.* The levels of both hs-TnT and hs-TnI were within the reference range and there was no significant (*P* > 0.05) difference between patients of the three groups. The hs-CRP values were higher in A-ED men compared with NA-ED (*P* = 0.048) but not compared with BL-ED (*P* = 0.136) and negatively correlated with IIF-5 (*r* = −0.480; *P* = 0.031). *Conclusions.* In ED patients of the three groups the measurement of hs-Tn allows us to exclude the presence of cardiac involvement at least when the history of ED is less than one year and the men are without atherosclerotic risk factors.

## 1. Introduction

Erectile dysfunction (ED) is now considered an early manifestation of a largely subclinical systemic vascular disorder affecting also the penile arteries. Indeed, ED shares with other vascular diseases, mainly coronary artery disease (CAD), common risk factors [1–4] and a similar pathogenic involvement of the NO pathway that leads to early impairment of endothelium-dependent vasodilatation and late obstructive vascular changes [5–8].

Cardiac troponin T (TnT) and troponin I (TnI) are the markers recommended for diagnosis of acute myocardial infarction [9]. With the implementation of assays with improved analytical sensitivity (hs-Tn), the reliable determination of troponin, even at concentrations below the previously defined 99th percentile values, has become possible. The limit of detection for the newest generation of hs-Tn assay is 10- to 100-fold lower than that of traditional assays [10]. Although an increase in troponin concentration classically indicates myocyte necrosis, several cellular processes other than necrosis can induce troponin release. Among them is increased cell wall permeability which may occur because of ischemia [11]. Test-induced ischemia has been associated with a quantifiable increase of troponin detectable with an ultrasensitive assay in proportion to the grade of ischemia, that is, mild or moderate to severe [12]. In addition to providing diagnostic information, the troponin levels offer powerful prognostic information even if detected in asymptomatic adults and moreover several cardiovascular risk factors have been shown to correlate with hs-Tn levels [13]. The emerging awareness of ED as an early warning for vascular health and possibly of silent CAD represents a unique opportunity to improve preventive cardiovascular health in all adult men who have persistent difficulty in achieving or maintaining an erection [14]. However, it is vital for effective management of the condition to isolate from this group of men those at high risk of CAD in particular to avoid treating those individuals who are not at risk of CAD and therefore who do not need it. The aim of our investigation was the early detection of cardiac injuries, even where no symptoms were present, using hs-Tn assays, in patients who have had ED symptoms for a few months, more than three months but less than one year, without known CAD risk factors. In fact, these patients are particularly susceptible to the development of CAD and would benefit the most from lifestyle modification or appropriate medical management earlier.

## 2. Material and Methods

Out of a series of 590 filed ED cases studied in the period of October 2009 to June 2011, we included in this study all patients with a history of ED of more than 3 months but less than one year. Exclusion criteria were clinical evidence from the patient's clinical history of coronary artery disease, dyslipidemia, arterial hypertension, malignancies, renal failure, congestive heart failure, systemic inflammatory disease, rheumatic disease, infection diseases, anemia, chronic liver diseases, arrhythmias, trauma, and no current smoking and vitamins supplementation or chronic drug assumption [15–19]. This investigation conforms to the principles outlined in the Declaration of Helsinki. Signed informed written consent was obtained from all subjects before their participation in the study. No ethical committee approval was required because no additional blood was needed for this study, and this was explained thoroughly to all patients, and also because this procedure has been reported to be acceptable [20–23].

On the basis of the given inclusion and exclusion criteria reported above, we selected a total of 120 ED patients who were included in the study. Their clinical characteristics are summarized in Table 1. The ED was classified as arteriogenic origin in 40 patients and as nonarteriogenic origin in 48 patients and, finally, in 32 patients the ED was classified as borderline. In our center, patients complaining of ED are currently investigated by careful history-taking and clinical andrological examination, then, a few days later, by a panel of examinations, including blood tests such as hemoglobin, glycated hemoglobin, glycemia, creatinine, high-sensitivity C-reactive protein (hs-CRP), total and HDL cholesterol, triglycerides, transaminases, testosterone, prolactin, 17-*β*-estradiol, urinalysis and 24 h urinary albumin excretion (microalbuminuria), the International Index of Erectile Function questionnaire (IIEF), and echo-color-Doppler of both cavernous arteries.

The IIEF questionnaire [24] is a validated, self-administered tool, but we only evaluated the answers to the first five (erectile response dominium) of the fifteen questions (IIEF-15, 1–5) [24, 25]. Possible scores for the IIEF-5 range from 5 to 25; scores of 22–25 indicate normal erectile function while scores of 21 or below indicate ED [25]. Penile echo-color-Doppler was done in basal conditions and after intracavernous injection of 10 *μ*g prostaglandin E1 (PgE1), and the peak systolic velocity (PSV) and end-diastolic velocity (EDV) were recorded 5, 10, 15, 20, and 25 min after the injection in the proximal portion of the penis. Patients were classified as “nonarteriogenic” (NA-ED) when their PSV was** ≥**35 cm/sec or ≤35 cm/sec but >25 cm/sec with concomitant EDV ≤0 cm/sec, “arteriogenic” (A-ED) when their PSV was ≤20 cm/sec, and “borderline” (BL-ED) with PSV between 25 and 21 cm/sec or with PSV between 35 and 25 with concomitant EDV >0 cm/sec [20–23, 26]. The erection quality was estimated 20 min after each injection. If a patient appeared stressed, he was given a second injection of the same dose of PgE1 and all measurements were repeated. Immediately before the penile echo-color-Doppler, participants were placed in a supine position and blood samples were drawn from a cubital vein. Samples were centrifuged at 3000 rpm, for 10 min. The serum was separated and stored at −80°C until analysis. All hs-TnT and hs-TnI were measured at the same time.

Serum concentrations of hs-TnT (Troponin T immunoassay; Roche Diagnostics, Switzerland) and of hs-TnI (Troponin I immunoassay; Johnson & Johnson, Italy) were measured according to the manufacturer's recommendations. The 99th percentile value for our normal reference population was 18.3 pg/mL for hs-TnT and 37 pg/mL for hs-TnI measured with a CV <10%.

All patients were referred to a cardiologist for evaluation of ischemic heart disease (IHD). The evaluation consisted of a comprehensive clinical history, physical examination, and an electrocardiogram while resting and during treadmill exercise. The diagnosis of ischemic heart disease relied on the detection of a 1 mm or more horizontal or downsloping ST-segment depression, frequent ventricular premature beats, or typical chest pain during the treadmill exercise test. In addition, the Duke Treadmill Score (DTS) as developed by Mark et al. [27] was calculated. The categories of risk based on the DTS are low risk (DTS > +5), moderate risk (DTS −10 to +4), and high risk (DTS < −11) [28].

All the results are expressed as the mean ± standard deviation. The data were not normally distributed and were normalized by using the log or log-log transformation. Undetectable values of hs-TnT and hs-TnI concentrations were considered equal to 0 for statistical purpose. In this regard, undetectable concentrations of hs-TnT were present in 49 out of 120 patients studied (40.8%) and undetectable concentrations of hs-TnI in 78 of the 120 studied patients (65.0%). The statistical analysis was carried out by using the ANOVA test taking into account the three groups of patients. The association between IIEF-5 and laboratory parameters was examined by Pearson's correlation coefficient. The estimate beta-power was equal to 0.004. *P* value < 0.05 was considered significant.

## 3. Results

Of the 120 men included in the study, 40 patients (33.3%) were classified as having A-ED, 48 patients (40%) as having NA-ED, and, finally, 32 patients (26.6%) as having BL-ED. In A-ED (mean age 52 ± 8.5 years), NA-ED (mean age 58.1 ± 7.3 years), and BL-ED (mean age 50.0 ± 10.5 years) the mean of IIEF values was 12.8 ± 2.4, 10.5 ± 2.6, and 11.6 ± 1.8, respectively. There were no significant differences between the three groups (*P* > 0.05). All the men had DTS > +5 without significant difference between the three groups (*P* > 0.05).

All our data of hs-TnT and hs-TnI concentrations were in the reference range, as reported in Table 1. No significant difference (*P* > 0.05) of serum concentration of both hs-TnT and hs-TnI was found among the three groups of ED patients. The hs-CRP values were higher in A-ED men compared with NA-ED (*P* = 0.048) but not compared with BL-ED (*P* = 0.136), while no significant difference was found between BL-ED and NA-ED (*P* = 0.964). Spearman's correlation analysis demonstrated a negative correlation between serum hs-CRP and IIEF-5 that reached the significance in A-ED (*r* = −0.480; *P* = 0.031) but without reaching the significance in NA-ED and BL-ED [29, 30]. No statistically significant differences were found regarding the other laboratory parameters evaluated (i.e., creatinine, glucose, ALT, AST, total cholesterol, HDL cholesterol, LDL cholesterol, triglycerides, HbA1c, 17-*β*-estradiol, prolactin, and testosterone) (Table 1).

## 4. Discussion

A consensus exists to consider ED an independent predictor of CVD [31–36], including CAD [37], peripheral artery disease [38], and stroke [39]. The early symptom of erectile difficulty often occurs before the development of structural, occlusive arterial disease and may therefore be one of the first signs of systemic vascular disease [14]. In other words, ED could be a sentinel marker for the presence of silent vascular disease in asymptomatic subjects. For this reason every effort should be made to check if cardiovascular risks are present in patients with ED or recognize when they begin to be present. Clinical studies revealed that the onset of ED symptoms occurs 2 to 3 years before CAD symptoms [40, 41] and 3 to 5 years before cardiovascular events [42, 43]. This relatively long time lag offers important potential in estimating and, ultimately, reducing cardiovascular risk in men with ED. However, it should be stressed that the penis is not always the most susceptible organ to inflammatory and atherosclerotic changes. Accordingly, although ED frequently precedes the onset of CAD, a considerable proportion of patients have CAD without concomitant ED, and vice versa, proving that the clinical course of atherosclerosis is multifaceted and not fully predictable. In diseases like ED, there is a need for a marker with a high negative predictive value for cardiac injury (ability to “rule out” the disease in patients with ED based on high sensitivity). In other words, it is imperative that a biomarker should ensure that ED cases at high risk of future cardiac events are not missed, because of the great benefit of treating these patients with a proven safe treatment. Cardiac troponins I and T are considered the most sensitive and specific biochemical markers of myocardial damage and the increased analytical sensitivity of the new generation methods demonstrated that measurable troponin was also present in the blood of almost 100% of healthy adults [44–46].

The aim of the study was to assess the presence of cardiovascular involvement with the measurement of both hs-TnT and hs-TnI levels in ED patients in order to begin the appropriate treatment quickly following the first symptoms to prevent significant cardiovascular events.

The populations studied were homogeneous without clinical evidence of atherosclerotic diseases; all men had normal ECG without ST-segment depression in exercise stress test and the DTS > +5.

Our results, using the measurement of hs-TnT or hs-TnI, at least in our experimental conditions, exclude the presence of cardiovascular injury in all patients with ED for less than one year and also in those of arteriogenic etiology. The fact that no differences were found among the three groups excludes the possibility of other statistical analyses such as the exploration of an optimal cut-point to differentiate the three groups.

In our patients we found that the serum concentrations of hs-CRP are higher in A-ED and the levels are associated with penile arterial disease severity assessed with IIEF-5. These data agree with those previously reported [29, 46–48]. In particular, plasma levels of hsCRP were found significantly higher in patients with ED and furthermore associated with penile arterial disease severity in men with ED without clinically apparent cardiovascular disease [30].

The number of patients included in this study seems to be low, but it must be considered that the population studied was carefully selected. In addition, the patients studied had no clinical evidence of atherosclerotic diseases and were free of the common risk factors associated with generalized penile arterial insufficiency such as hypertension, hyperlipidemia, cigarette smoking, diabetes mellitus, and pelvic irradiation. This is the first study to compare the value of troponin as a marker of cardiovascular disease before clinical symptoms in ED patients. Finally, we do not know whether troponin (T or I) may add information by adding the determination of other biomarkers such as NT-proBNP, as recently suggested [49, 50], or clinical variables such as echocardiography. Given the complementary and independent prognostic value of various markers, a “multimarker” approach in men with ED may be an effective strategy to improve prediction of cardiovascular risk beyond the use of traditional risk factors in daily clinical practice.

In conclusion, this study shows that the measurement of hs-TnT or hs-TnI levels allows us to exclude the presence of cardiovascular disease in patients with ED. The reason is probably the short history of ED, which was of less than one year. Longitudinal study is currently in progress to follow the level of hs-Tn (I and T) in this selected group of men, particularly in A-ED, in order to recognize when the heart begins to be affected by the process of atherosclerosis, before clinical symptoms. At present, we know the basal levels of both hs-Tn types in all our patients, and knowing the reference change values (RCV) [51–54], we can recognize when the level will increase over the RCV, even if it remains within the reference interval. Knowing that a minimum clinical change if present would indeed be diagnostically useful.

## Figures and Tables

**Table 1:** Clinical and laboratory features of patients with erectile dysfunction. Values are reported as mean and standard deviation.

	NA-ED (*n* = 40)	A-ED (*n* = 48)	BL-ED (*n* = 32)
Patients data			
Age (years)	52.0 ± 8.5	58.1 ± 7.3	50.0 ± 10.5
IIEF-5 score	12.8 ± 2.4	10.5 ± 2.6	11.6 ± 1.8
Laboratory evaluation			
Study parameters			
hs-cTnT (pg/mL)	2.89 ± 5.01	4.32 ± 7.29	3.10 ± 4.18
hs-cTnI (pg/mL)	4.72 ± 10.93	2.4 ± 6.25	0.79 ± 3.88
hs-CRP (mg/L)	1.70 ± 1.78	3.09 ± 3.19^*^	2.03 ± 2.79
Other parameters			
Creatinine (mg/dL)	0.92 ± 0.16	0.95 ± 0.16	0.96 ± 0.15
Glucose (mg/dL)	102.9 ± 6.3	93.0 ± 14.1	95.6 ± 10.8
ALT (U/L)	26.22 ± 15.76	23.10 ± 10.29	24.46 ± 8.29
AST (U/L)	23.56 ± 10.16	21.60 ± 5.71	27.29 ± 10.28
Total cholesterol (mg/dL)	200.36 ± 10.21	207.80 ± 11.72	205.21 ± 9.49
HDL cholesterol (mg/dL)	55.14 ± 13.90	50.30 ± 10.19	52.46 ± 15.82
LDL Cholesterol (mg/dL)	122.56 ± 11.53	133.60 ± 10.68	128.04 ± 10.40
Triglycerides (mg/dL)	112.97 ± 62.23	123.90 ± 58.60	125.54 ± 59.02
HbA1c (%)	5.27 ± 0.50	5.77 ± 0.52	5.56 ± 0.44
17-ß-Estradiol (pg/mL)	27.23 ± 8.29	29.17 ± 8.61	27.45 ± 10.57
Prolactin (ng/mL)	8.59 ± 4.66	8.64 ± 3.43	9.23 ± 3.52
Testosterone (ng/mL)	4.97 ± 2.50	4.13 ± 1.64	4.59 ± 1.29

NA-ED: nonarteriogenic erectile dysfunction; A-ED: arteriogenic erectile dysfunction; BL-ED: borderline arteriogenic erectile dysfunction.

^*^ *P* < 0.048 versus NA-ED.
